# Evaluation of the Coulter Counter S-Plus VI

**DOI:** 10.1155/S1463924688000094

**Published:** 1988

**Authors:** Giuseppe Banfi, Marina Pontillo, Paola Notti, Pierangelo Bonini

**Affiliations:** Istituto Scientifico S. Raffaele Laboratorio Analisi Via Olgettina 60 Milano Italy

## Abstract

This article reports an evaluation of the Coulter Counter model S-Plus VI automatic analyser for haematology, and data are presented on linearity, carry-over, precision, accuracy and stability of the instrument, when compared with a model S-Plus IV/D.

The three-part differential count provided by Coulter S-Plus VI has been compared with manual eye counting. The results show a good agreement with only 2.5% of discrepancies in 2271 routine samples.

Advantages of the new instrument include: reduction of running costs, largely due to manpower saving; simple and easy use, and improved operator safety, there being no need for human contact with blood.


Journal of Automatic Chemistry, Vol. 10, No. 1 (January–March 1988), pp. 31–36

### Evaluation of the Coulter Counter S-Plus VI

**Giuseppe Banfi, Marina Pontillo, Paola Notti and Pierangelo Bonini**

*Istituto Scientifico S. Raffaele, Laboratorio Analisi, Via Olgettina 60, Milano, Italy*

*This article reports an evaluation of the Coulter Counter model S-Plus VI automatic analyser for haematology, and data are presented on linearity, carry-over, precision, accuracy and stability of the instrument, when compared with a model S-Plus IV/D.*

*The three-part differential count provided by Coulter S-Plus VI has been compared with manual eye counting. The results show a good agreement with only 2.5% of discrepancies in 2271 routine samples.*

*Advantages of the new instrument include: reduction of running costs, largely due to manpower saving; simple and easy use, and improved operator safety, there being no need for human contact with blood.*

### Introduction

Performance of automatic instruments for haematology has been considerably improved in recent years, resulting in increased instrument throughput (number of samples processed), a reduction in the volume of sample required, and a simpler and more standardized interface with EDP.

There are two crucial aspects of automation in haematology: sampling and differential counting. The advantages to automating sampling include a reduction of manual work at a tedious phase in the procedure where risks of sample mismatching is high; standardization in sample mixing; and, most importantly, a reduction in the risks of operator infection. This is especially so if a closed system with automatically pierceable caps is used. Unfortunately, the need to maintain sample homogeneity, while avoiding haemolysis in the pre-aspiration and aspiration phases, creates so many technical problems that in most commercial instruments, sampling is manual.

Differential counting has been automated using either computer-controlled pattern recognition or by means of rather complicated and expensive cytochemical reactions.

Two new automatic haematology instruments have been developed recently by Coulter (Models S-Plus IV/D and S-Plus VI). These are able to perform, in addition to the traditional haemocytometric measurements, a simplified differential count with only three parameters instead of the classical five, using a sample size as low as 100 µl. The Coulter S-Plus VI is also provided with an automatic sampler (CASH), containing up to 32 tubes and with a throughput of 90 samples per hour. In the Coulter S-Plus IV/D sampling is manual. An evaluation of these instruments is presented in this paper; particular attention has been given to the innovative aspects connected with differential counting and sampling.

* Correspondence to Dr Bonini.

### Materials and methods

The Coulter S-Plus IV/D is an automated multiparameter hematology analyser, based on the Coulter principle of impedence counting. Like most of the previous S-Plus models it has two counting chambers, operating simultaneously, one for platelets and erythrocytes and the other for leukocytes. Each is provided with three apertures, for triplicate analyses.

Differential counting is done by means of a special lysing reagent, which, in addition to completely haemolysing erythrocytes in the appropriate counting chamber, provokes a shrinking of the leukocyte cytoplasm; consequently, lymphocytes are reduced to one half their original volume while granulocytes, because of their cytoplasmic granules, shrink to only two-thirds of their original size [2 and 3]. A third group of cells (monocytes and other mononuclear cells) have volumes in between those of lymphocytes and granulocytes. These three classes of artificially modified leukocytes are then counted in the white cell chamber and differentiated on the basis of their 'apparent' volume.

Three histograms show the size distribution for leukocytes, erythrocytes and platelets respectively. The leukocytes are categorized into three subpopulations: (1) lymphocytes with volumes between 35–90 fL and claimed to include mature lymphocytes, atypical lymphocytes and lymphoma cells; (2) mononuclear cells with volumes between 90 and 160 fL, claimed to include monocytes, blasts, promyelocytes and myelocytes; (3) granulocytes with volumes between 160 and 450 fL and including polymorphonuclears, bands, metamyelocytes and basophils [2 and 3]. Whether or not to include eosinophils in the class of mononuclear cells and/or granulocytes is a much debated point.

This classification of leukocytes is based on the analysis of some 20 000 cells. When the valleys between the three subpopulations are not clearly defined in the histogram, the instrument produces a flag. An R1 flag indicates the presence of particles smaller than 35 fL, which can be debris of cells, platelet clumps, erythroblasts, or non-lysed erythrocytes, all producing a 'high take-off' of WBC histogram. A 'backlighting' of absolute values of WBC and of Ly, Mo, Gr also appears.

An R2 flag indicates abnormal and/or immature lymphocytes, monocytosis, reactive lymphocytosis, eosinophilia and/or blasts. An R3 flag indicates high neutrophilia and/or abnormal granulocytes. A combination of the above anomalies produces a RM flag.

The Coulter S-Plus VI differs from the Coulter S-Plus IV/D in being provided with an automatic sampler (CASH) – a mechanical device which simultaneously operates two rotating trays. Up to 32 specimens, in addition to the control 4C Plus, may be inserted in each tray.

A Coulter S-Plus VI has been installed in our laboratory for the present evaluation, and operated for five weeks according to the manufacturer's instructions, alongside an already operating Coulter S-Plus IV/D. The evaluation has been performed according to the ICSH protocol [7].

An internal quality control programme operates daily using the 4C Plus blood control and checking the moving averages for various parameters. An external quality control programme (Ortho Diagnostic Systems, Milan, Italy) operates at two levels: normal and abnormal.

Samples were collected in vacuum tubes (Vacutainer, Becton Dickinson, Milan, Italy) of 3 ml capacity containing K3EDTA as anticoagulant, and, except for the study of precision and stability, processed between 1 and 5 h from drawing.

For the study of linearity, plasma and blood cells separated after centrifugation from five routine specimens were mixed in different proportions and the resulting mixtures analysed in the Coulter S-Plus VI. Within-run precision was evaluated by carrying out 10 determinations on five routine specimens. Between-run and overall precision was calculated from 10 determinations done on one normal specimen every 60 min, up to 8 h after venipuncture, and finally after 24 h. The blood was stored at room temperature. The same data have been used to evaluate the stability of a sample after blood drawing. To evaluate the stability of the instrument, measurements obtained during 19 working days on the same lot of 4C Plus control material were recorded and compared with the assigned values. Carry-over was evaluated on a routine sample tested in duplicate, before and after three aspirations of diluent (Isoton III); the same experiment was repeated on the same sample, after centrifugation and partial removal of plasma.

For checking the accuracy of differential counting, results from the Coulter S-Plus VI were compared with those from (1) a reference method consisting of a microscopic examination by a well-trained operator, of smears stained with May Grunwald-Giemsa, and differential counting on 500 cells: 50 samples were examined in this way; (2) a routine method, consisting of a microscopic examination of smears stained with May Grunwald-Giemsa, and differential counting on 100 cells. These measurements were performed on all the laboratory routine samples (2271 in total) for five weeks.

In neither case did the operators know the results obtained with the Coulter S-Plus VI.

To compare the overall performance of the Coulter S-Plus VI and Coulter S-Plus IV/D, 150 random samples from inpatients and out-patients were tested with both instruments running under routine conditions, with normal quality control and manufacturer's calibration.

### Results

*Linearity*

Plasma and blood cells from five routine samples have been mixed in different proportion and the following extreme values were obtained: 0·1–15·4 × $10^9$/l for WBC, 0·1–10·6 × $10^{12}$/l for RBC, 10–473 × $10^9$/l for PLT, 0–28·7 g/dl for Hb, 0–87·3% for Hct. The observed values of WBC, RBC, Hct, PLT and Hb have been plotted against the expected values (figure 1); the linearity is excellent for all the parameters within the ranges examined (WBC: $y = -0{\cdot}61 + 1{\cdot}04x$, $r = 0{\cdot}996$; Hb: $y = 0{\cdot}41 + 0{\cdot}99x$, $r = 0{\cdot}999$; Hct: $y = 0{\cdot}64 + 0{\cdot}99x$, $r = 0{\cdot}999$; RBC: $y = -0{\cdot}12 + 1{\cdot}02x$, $r = 0{\cdot}994$; PLT: $y = 26{\cdot}8 + 0{\cdot}96x$, $r = 0{\cdot}994$). As figure 2 illustrates, a certain elevation of lymphocytes, and a corresponding reduction of granulocytes, occurs when the ratio between blood cells and plasma is lowered, while a rather irregular pattern of monocytes can be observed. The same occurs also when haematocrit is kept constant while diluting leukocytes in plasma. This phenomenon is probably due to a modification of some physico-chemical properties of WBC due to centrifugation and/or plasma removal as it is suggested by the lack of correspondence between the true values of each category of leukocytes and the values observed at a sample dilution corresponding to the original (before dilution) haematocrit (figure 2). If this is the case, this fact is irrelevant in terms of the quality of results, as there is no need for such manipulations when performing routine haematology tests. Further investigation is needed.

*Precision*

An excellent level of precision, even better than manufacturer's claims, is illustrated in tables 1 and 2. The only

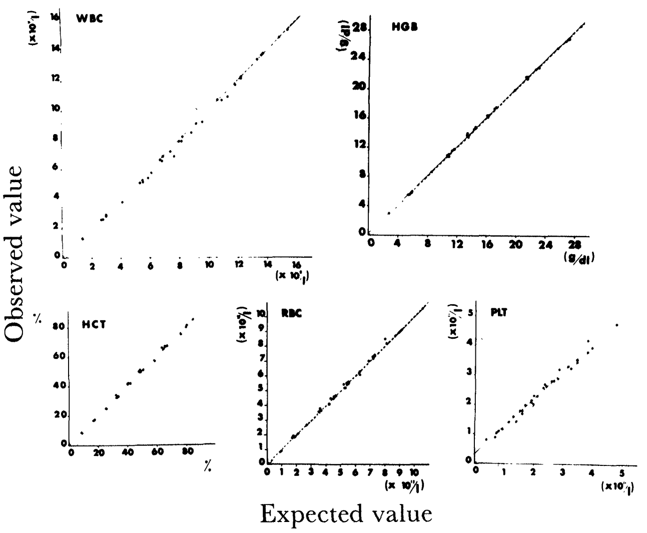

*Figure 1. Correlation between expected and observed values for leukocytes, haemoglobin, haematocrit, erythrocytes and platelets in a linearity test.*

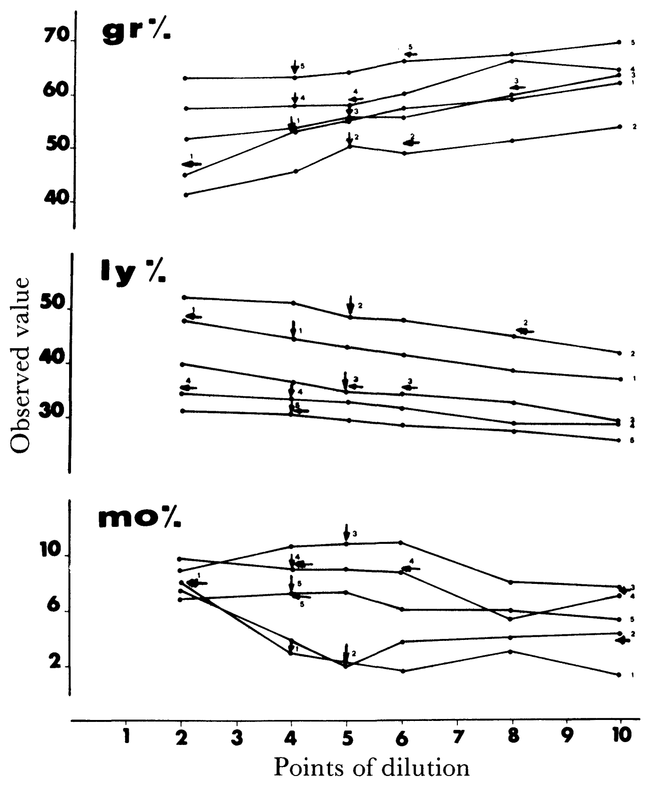

*Figure 2. Values of the three parameters differential count in the five samples studied for linearity, indicated with the numbers from 1 to 5, at different dilutions. Vertical arrows indicate the dilution corresponding to the native haematocrit; horizontal arrows indicate the native value of each class of leukocytes for each sample; in some cases, when values identical to native one is obtained at more than one dilution, more than one horizontal arrow is reported (see Gr% and Ly% for patient n. 3 and Mo% for patient n. 4). Obviously, for each sample, the horizontal and vertical arrows were expected to indicate the same point, which was not the case in most samples.*

exception is mononuclear cells where an overall coefficient of variation of 10% has been observed.

### *Stability*

The results of stability test on one sample presented a good stability until 6–7 h after blood drawing for all parameters, except MPV, where some 6–7% of increase is observed just after 1 h, and Mo% which presents a wide range of percent variation. The values observed on the 4C Plus during 19 working days (table 3) are within the assigned ranges and are very stable.

### *Carry-over*

Practically no carry-over has been observed, even in very extreme conditions as in a concentrated sample with the following values: 18·3 × $10^9$/l for WBC, 8·75 × $10^{12}$/l for RBC, 372 × $10^9$/l for PLT and 28·1 g/dl for Hb. After one measurement cycle on this concentrated sample, an analysis performed on the diluent Isoton III gave these results: 0·1 × $10^9$/l for WBC, 0·04 × $10^{12}$/l for RBC, 3 × $10^9$/l for PLT, 0·1 g/dl for Hb. Two subsequent determinations performed on the same Isoton III did not present a background for WBC, RBC, Hb and a negligible one in the PLT count (1 × $10^9$/l).

### *Comparisons*

An excellent correlation exists between the results obtained with Coulter S-Plus VI and Coulter S-Plus IV/D (figures 3[*a*] and 3[*b*]) with a very limited dispersion of data for all the haematologic parameters except for mononuclear cells, which show some dispersion around the correlation line. The following regression equations and coefficients were observed: WBC: $y = 0{\cdot}9x$, $r = 0{\cdot}999$; RBC: $y = 1{\cdot}01x$, $r = 1$; PLT: $y = 1{\cdot}43 + 1{\cdot}15x$, $r = 0{\cdot}998$; Hb: $y = 1{\cdot}01x$, $r = 1$; Hct: $y = 0{\cdot}02 + 0{\cdot}98x$, $r = 0{\cdot}999$; MCV: $y = -0{\cdot}001 + 1{\cdot}058x$, $r = 0{\cdot}999$; RDW:

*Table 1. Within-run precision on six routine samples.*

| | Within-run precision | | | | | | | | | | | | | | | | | |
|---|---|---|---|---|---|---|---|---|---|---|---|---|---|---|---|---|---|---|
| Samples | x̄ | SD | CV % | x̄ | SD | CV % | x̄ | SD | CV % | x̄ | SD | CV % | x̄ | SD | CV % | x̄ | SD | CV % |
| Wbc (×$10^9$/l) | 9·18 | 0·06 | 0·69 | 4·48 | 0·06 | 1·40 | 7·62 | 0·09 | 1·18 | 5·91 | 0·07 | 1·23 | 6·52 | 0·08 | 1·21 | 6·54 | 0·05 | 0·79 |
| Rbc (×$10^{12}$/l) | 4·63 | 0·01 | 0·25 | 3·41 | 0·02 | 0·58 | 4·44 | 0·02 | 0·40 | 3·56 | 0·02 | 0·48 | 4·87 | 0·04 | 0·82 | 4·79 | 0·02 | 0·54 |
| Hb (g/dl) | 14·27 | 0·06 | 0·47 | 8·45 | 0·05 | 0·63 | 13·42 | 0·08 | 0·58 | 11·06 | 0·05 | 0·47 | 15·20 | 0·13 | 0·85 | 14·37 | 0·08 | 0·57 |
| Hct (%) | 41·96 | 0·24 | 0·57 | 27·44 | 0·22 | 0·80 | 41·54 | 0·24 | 0·58 | 33·42 | 0·19 | 0·57 | 44·69 | 0·29 | 0·65 | 43·34 | 0·23 | 0·53 |
| Mcv (fl) | 90·57 | 0·50 | 0·55 | 80·36 | 0·53 | 0·66 | 93·57 | 0·35 | 0·37 | 93·95 | 0·26 | 0·28 | 91·70 | 0·42 | 0·46 | 90·52 | 0·26 | 0·29 |
| Mch (pg) | 30·81 | 0·16 | 0·51 | 24·75 | 0·26 | 1·03 | 30·22 | 0·22 | 0·73 | 31·09 | 0·25 | 0·80 | 31·17 | 0·17 | 0·54 | 30·00 | 0·12 | 0·40 |
| Mchc (g/dl) | 34·02 | 0·26 | 0·76 | 30·76 | 0·31 | 1·02 | 32·30 | 0·29 | 0·90 | 33·09 | 0·25 | 0·75 | 33·99 | 0·20 | 0·59 | 33·15 | 0·10 | 0·30 |
| Rdw (%) | 12·77 | 0·24 | 1·89 | 20·33 | 0·35 | 1·72 | 12·02 | 0·20 | 1·66 | 14·38 | 0·44 | 3·06 | 12·83 | 0·12 | 0·90 | 12·82 | 0·17 | 1·32 |
| Plt (×$10^9$/l) | 339·10 | 8·14 | 2·40 | 246·40 | 4·14 | 1·67 | 298·00 | 4·84 | 1·62 | 271·50 | 3·57 | 1·31 | 158·00 | 3·71 | 2·30 | 217·80 | 4·39 | 2·01 |
| Pct (%) | 0·245 | 0·007 | 2·85 | 0·203 | 0·002 | 0·13 | 0·265 | 0·005 | 1·88 | 0·254 | 0·008 | 3·15 | 0·123 | 0·003 | 2·40 | 0·197 | 0·005 | 2·54 |
| Mpv (fl) | 7·23 | 0·09 | 1·31 | 8·25 | 0·12 | 1·43 | 8·88 | 0·10 | 1·13 | 9·30 | 0·20 | 2·15 | 7·82 | 0·10 | 1·28 | 9·08 | 0·12 | 1·32 |
| Pdw (%) | 16·68 | 0·25 | 1·49 | 16·99 | 0·28 | 1·63 | 16·15 | 0·16 | 0·98 | 16·57 | 0·26 | 1·57 | 16·35 | 0·20 | 1·22 | 16·81 | 0·19 | 1·13 |
| Ly (%) | 40·37 | 0·74 | 1·84 | 26·74 | 0·80 | 2·99 | 30·07 | 0·42 | 1·43 | 35·60 | 0·60 | 1·64 | 37·96 | 1·22 | 3·20 | 31·57 | 0·45 | 1·42 |
| Mo (%) | 5·57 | 0·49 | 6·79 | 9·07 | 0·57 | 6·28 | 7·79 | 0·67 | 7·70 | 8·27 | 1·13 | 13·66 | 7·35 | 0·57 | 7·75 | 8·06 | 0·66 | 8·18 |
| Gr (%) | 54·06 | 1·06 | 1·96 | 64·19 | 1·12 | 1·74 | 62·14 | 0·84 | 1·35 | 55·23 | 1·48 | 2·68 | 54·31 | 1·36 | 2·50 | 60·37 | 0·71 | 1·17 |
| Ly (×$10^9$/l) | 3·71 | 0·05 | 1·34 | 1·21 | 0·03 | 2·48 | 2·29 | 0·06 | 2·49 | 2·16 | 0·05 | 2·36 | 2·51 | 0·09 | 3·46 | 2·06 | 0·05 | 2·42 |
| Mo (×$10^9$/l) | 0·52 | 0·04 | 7·69 | 0·40 | 0 | 0 | 0·59 | 0·06 | 9·66 | 0·49 | 0·09 | 1·77 | 0·48 | 0·04 | 8·70 | 0·51 | 0·05 | 9·80 |
| Gr (×$10^9$/l) | 4·97 | 0·08 | 1·60 | 2·88 | 0·09 | 3·19 | 4·72 | 0·10 | 2·11 | 3·27 | 0·08 | 2·50 | 3·55 | 0·08 | 2·40 | 3·95 | 0·08 | 2·02 |

*Table 2. Overall precision on one routine sample.*

| | Overall precision | | | | | |
|---|---|---|---|---|---|---|
| | X | SD | CV% within run | CV% between run | CV% overall | Total CV% |
| Wbc (× $10^9$/l) | 8·90 | 0·220 | 1·57 | 2·47 | 2·92 | 2·50 |
| Rbc (×$10^{12}$/l) | 4·57 | 0·099 | 0·65 | 2·17 | 2·28 | 2·17 |
| Plt (×$10^9$/l) | 322·58 | 11·500 | 2·30 | 3·49 | 4·18 | 3·56 |
| Hb (g/dl) | 14·10 | 0·195 | 2·86 | 1·00 | 3·05 | 1·38 |
| Hct (%) | 41·50 | 0·936 | 0·70 | 2·24 | 2·36 | 2·25 |
| Mcv (fl) | 91·40 | 0·370 | 0·53 | 0·36 | 0·63 | 0·41 |
| Rdw (%) | 12·78 | 0·335 | 1·37 | 2·58 | 2·89 | 2·62 |
| Mpv (fl) | 7·90 | 0·490 | 1·17 | 6·20 | 6·32 | 6·20 |
| Gr (%) | 52·61 | 1·270 | 1·82 | 2·34 | 2·96 | 2·41 |
| Mo (%) | 6·67 | 0·450 | 7·79 | 6·29 | 10·00 | 6·86 |
| Ly (%) | 40·76 | 1·240 | 2·00 | 2·96 | 3·60 | 3·05 |

*Table 3. Mean, range and standard deviation values for WBC, RBC, Hb, Hct, MCV, PLT in one single lot of 4C Plus during 19 working days. Stability of 4C Plus – Lot 9635 – from 3 February 1986 to 24 February 1986.*

| | | Assigned value range | | Observed values Mean | Observed values Range | SD |
|---|---|---|---|---|---|---|
| Wbc | (×$10^9$/l) | 7·9 | ±4 | 7·79 | 7·4–8·2 | 0·20 |
| Rbc | (×$10^{12}$/l) | 4·24 | ±0·1 | 4·18 | 4·13–4·26 | 0·04 |
| Hb | (g/dl) | 13·1 | ±0·3 | 13·2 | 13–13·5 | 0·17 |
| Hct | (%) | 37·1 | ±1·5 | 36·84 | 36·4–37·5 | 0·36 |
| Mcv | (fl) | 87·5 | ±2 | 87·96 | 86·9–89·1 | 0·53 |
| Plt | (×$10^9$/l) | 211 | ±25 | 212 | 197–232 | 10·60 |

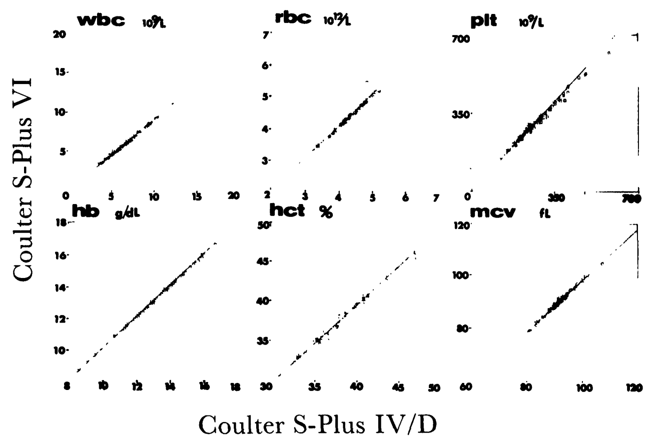

*Figure 3(a). Correlation between values obtained with Coulter S-Plus VI and Coulter S-Plus IV/D on 150 routine samples for WBC, RBC, PLT, Hb, Hct and MCV.*

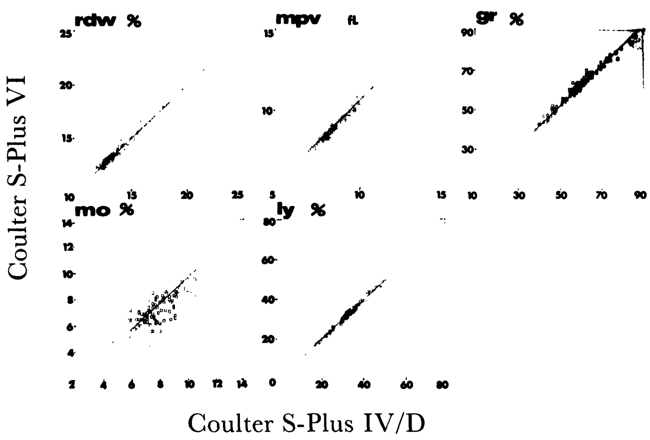

*Figure 3(b). Correlation between values obtained with Coulter S-Plus VI and Coulter S-Plus IV/D on 150 routine samples for RDW, MPV, Gr, Mo and Ly.*

$y = 0{\cdot}002 + 0{\cdot}99x$, $r = 0{\cdot}999$; MPV: $y = -0{\cdot}001 + 1{\cdot}058x$, $r = 0{\cdot}999$; Gr: $y = 0{\cdot}70 + 1{\cdot}02x$, $r = 0{\cdot}999$; Mo: $y = 0{\cdot}025 + 0{\cdot}96x$, $r = 0{\cdot}990$; Ly: $y = -0{\cdot}01 + 0{\cdot}96x$, $r = 0{\cdot}999$. The correlation between the microscopic observation by a well-trained personnel on 500 cells on one side, and results from Coulter S-Plus VI and Coulter S-Plus IV/D from the other one is rather poor for mononuclear cells; it is very good, however, for granulocytes and lymphocytes (figure 4). The following regression equations and coefficients were observed for Coulter S-Plus IV/D versus microscopic observation: Ly%: $y = 2{\cdot}95 + 0{\cdot}97x$, $r = 0{\cdot}954$; Mo%: $y = 2{\cdot}92 + 0{\cdot}32x$, $r = 0{\cdot}259$; Gr%: $y = 4{\cdot}38 + 0{\cdot}93x$, $r = 0{\cdot}952$ and for Coulter S-Plus VI versus microscopic observation: Ly%: $y = 3 + x$, $r = 0{\cdot}955$; Mo%: $y = 2{\cdot}52 + 0{\cdot}39x$, $r = 0{\cdot}292$; Gr%: $y = 2{\cdot}47 + 0{\cdot}94x$, $r = 0{\cdot}944$.

Comparison between differential countings from Coulter S-Plus VI and routine microsccopic observation over a five-week period (table 4) shows that, despite the

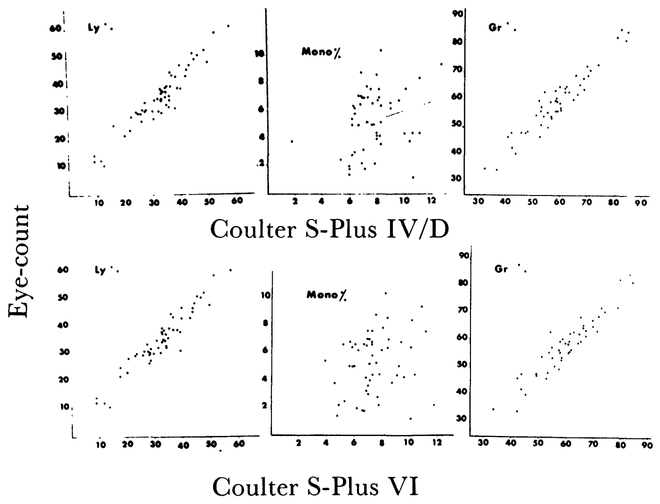

*Figure 4. Correlation between values obtained with, respectively, Coulter S-Plus IV/D (upper part) and Coulter S-Plus VI (lower part) and microscopic observation.*

*Table 4. Correlation between differential counting obtained with the Coulter S-Plus VI and routine microscopic examination.*

| | | |
|---|---|---|
| | Total number of considered differential counts | 2271 |
| 1. | Discrepancies between differential counts from Coulter VI and from eye-count (100 cells) | 363 (16%) |
| | of which | |
| 2. | Discrepancies due to eosinophilia | 295 (13%) |
| 3. | Significant discrepancies between differential counts from Coulter VI and from eye-count (100 cells), according to the Rumke's table | 54 (2·37%) |
| 4. | Cases with flags or backlighting | 64 (2·8%) |
| 5. | Cases with dots or dashes (incomplete computation) | 16 (0·7%) |
| 6. | Cases correlation between those indicated in 3 (above) and 4 | 39 (72·22% of cases in 3) |
| 7. | False positives | 43 (1·9%) |
| 8. | False negatives | 16 (0·7%) |

apparently large number of discrepancies in results (16%), mainly due to eosinophilias (13%), only in 54 cases (2·4%), modifications of automatic differential counting after a microscopic observation of 100 cells are really significant, according to the Rumke's table [10] and most of these cases (72%) were flagged by the automatic instrument. Sensitivity (false negatives) and specificity (false positives) are 0·7 and 1·9% respectively.

### Discussion

It was emphasized in the Introduction that for haematology, automation of the pre-analytical phase (sampling) and differential counting are the crucial problems.

For sampling, the excellent correlation between data observed on the Coulter S-Plus VI and the Coulter S-Plus IV/D with the same samples, demonstrates that the adoption of CASH in the Coulter S-Plus VI resulted in good mixing of samples before their aspiration and produced many operational benefits, compared with manual sampling. These included: reduction of manual work and possible errors; reduced risk of infection because of the use of vacuum tubes and direct aspiration by the needle of the CASH, and standardization of sample mixing. The adoption of three-parameter differential counting, even if greatly modifying the classical five-parameter pattern, in our experience is acceptable both from an analytical and clinical point of view. Granulocyte and lymphocyte counts correlate very well with manual counting performed on 500 cells by a well-trained operator. This agrees with other data from the literature [1, 4 and 5]. The sophisticated system of flags indicating clinical abnormalities from the histogram patterns gives confidence for the adoption of this system – note, in table 4, the very low number of significant modifications of automatic differential counting necessary after checking by microscopic examination.

A smaller number of false positives and negatives were observed than reported by other authors [4 and 9]; this was probably due to the limited number of cases with abnormal haematology in our population. In agreement with other authors [1, 4 and 5] we also observed a poor correlation between automatically determined mononuclear cells and microscopic counting of monocytes; even the precision is poorer than those of granulocytes and lymphocytes and a greater dispersion of data is observed in comparing data of Coulter S-Plus VI with Coulter S-Plus IV/D, partially due to the low number of this category of cells.

As discussed by other authors [4 and 6], the mononuclear cells fraction, rather than corresponding to a well-defined category of cells, should be considered as a remainder fraction, including cells other than monocytes, such as blasts, promyelocytes and myelocytes. These must be identified, when necessary, under the microscope.

Important advantages of automatic differential counting are the greater reliability of results based on the examination of a much larger number of cells than is usually counted under the microscope (20 000 for Coulter against 100 for microscope), and the elimination of the fastidious and very repetitive microscopic examination of a large number of smears, many of which are normal in most laboratories.

In our laboratory, after the present evaluation, we have decided to limit microscopical differential counting to specimens where a flag for differential counting and/or alterations of an other haematologic parameter are present (approximately 30% of our routine work). In addition, the clinical pathologist obtains much clinically useful information from careful study of the histograms. This aspect will be reported in future papers. Overall, there is a considerable time saving for clinical pathologists. The clinician receives an haematologic report for each patient based on reliable differential counting of granulocytes and lymphocytes [8] and on a critical study of the Coulter histograms.

In summary, and in agreement with manufacturer's claims and data in literature, our findings confirm good linearity, stability (up to 8 h after drawing) and precision, with negligible carry-over. The instrument throughput, the very low sample size and reagent consumption have not been reported in detail but correspond to the claims of manufacturer. The instrument was easy to operate and was well accepted by the laboratory staff.

### References

1. Cornbleet, S. and Kessinger, S., *American Journal of Clinical Pathology,* **84** (1985), 620.
2. Coulter Electronics, *Hematology Analyzer,* **5** (1984), 1.
3. Coulter Electronics, *Product Reference Manual; S-Plus IV with 3-part differential and auto transfer* (Hialeah, Coulter Electronics, 1983).
4. Cox, C. J., Habermann, T. M., Payne, B. A., Klee, G. G. and Pierre, R. V., *American Journal of Clinical Pathology,* **84** (1985), 297.
5. Greendyke, R. M., Kanter, D. R., Deboover, L., Savage, L. and Vangelder, S., *American Journal of Clinical Pathology,* **84** (1985), 348.
6. Griswold, D. J. and Champagne, V. D., *American Journal of Clinical Pathology,* **84** (1985), 297.
7. International Committee for Standardization in Haematology (ICSH), *Clinical and Laboratory Haematology,* **6** (1984), 69.
8. Koepke, J. A., *Laboratory Medicine,* **11** (1980), 371.
9. Nelson, L., Charache, S., Keyser, E. and Metzger, P., *American Journal of Clinical Pathology,* **83** (1985), 547.
10. Rumke, C. L., in *Differential Leukocyte Counting.* Edited by Koepke, J. A. (Aspen/College of American Pathologists, 1977), 39.

### List of abbreviations

WBC or wbc = white blood cells; RBC or rbc = red blood cells; Hb or hb or HGB = haemoglobin; Hct or HCT or hct = haematocrit; PLT or plt = platelets; MCV or mcv = mean corpuscolar volume; mch = mean corpuscolar haemoglobin; mchc = mean corpuscolar haemoglobin concentration; RDW or rdw = red cell distribution width; MPV or mpv = mean platelet volume; pdw = platelet distribution width; pct = thrombocrit; Ly or ly = lymphocytes; Mo or mo or Mono = monocytes; Gr or gr = granulocytes.

### FIRST INTERNATIONAL SYMPOSIUM ON DRUGS IN COMPETITIVE ATHELETICS

*To be held in Yugoslavia from 29 May to 2 June 1988*

The symposium (which is sponsored by IUPAC and organized under the auspices of the IFCC) will assemble scientists and physicians of varying backgrounds with a common interest in toxicology and sports medicine. The symposium will be organized in a manner so as to facilitate discussion with exchange of information and ideas. There will be plenary sessions to introduce and review a topic, followed by open forum discussions of various aspects of such topics.

Plenary lectures include:

D. H. Catlin (USA) – Drugs in competitive athletics

D. A. Cowan (UK) – Specimen acquisition, quality assurance and chain of custody

R. Dugal (CND) – Analytical requirements for detection of drugs of abuse and anabolic steroids

H. W. Durbeck (FR Germany) – Gas chromatography – mass spectrometry procedures

R. Hampl (CS) – Endocrine effects and immunoassay procedures

A. Ljungquist (S) – Health risks of steroid use

S. Rendić (YU) – Identification of drug metabolites by gas chromatography – mass spectrometry

J. R. Shipe (USA) – Mass spectrometry instrumentation in the 1990s

S. D. Vesselinovitch (USA) – Synthetic steroids as potential carcinogens.

There will also be an open forum on drug testing at major athletic events. Principal organizers of drug testing for the Olympic Games held in Montreal (1976); Sarajevo (1984), Los Angeles (1984) and Universiade '87 Zagreb will participate as panel members in this open forum.

*Further information from either Professor M. Mikac-Dević, University Hospital 'Dr Mladen Stojanović', Department of Clinical Chemistry, 41000 Zagreb, Yugoslavia; or Dr John Savory, Box 168, Department of Pathology, University of Virginia Medical Center, Charlottesville, Virginia 22908, USA.*

